# Hemophilia a patients with inhibitors: Mechanistic insights and novel therapeutic implications

**DOI:** 10.3389/fimmu.2022.1019275

**Published:** 2022-12-08

**Authors:** Liping Luo, Qiaoyun Zheng, Zhenyu Chen, Meijuan Huang, Lin Fu, Jianda Hu, Qizhen Shi, Yingyu Chen

**Affiliations:** ^1^ Department of Hematology, Fujian Provincial Key Laboratory of Hematology, Fujian Institute of Hematology, Fujian Medical University Union Hospital, Fuzhou, Fujian, China; ^2^ Medical Technology and Engineering College of Fujian Medical University, Fuzhou, Fujian, China; ^3^ Department of Hematology, The Second Affiliated Hospital of Guangzhou Medical University, Guangzhou, Guangdong, China; ^4^ Department of Pediatrics, Medical College of Wisconsin, Milwaukee, WI, United States; ^5^ Blood Research Institute, Versiti, Milwaukee, WI, United States; ^6^ Children’s Research Institute, Children’s Wisconsin, Milwaukee, WI, United States; ^7^ Midwest Athletes Against Childhood Cancer (MACC) Fund Research Center, Milwaukee, WI, United States

**Keywords:** Hemophilia A, inhibitor, immune tolerance, pathogenesis, factor VIII

## Abstract

The development of coagulation factor VIII (FVIII) inhibitory antibodies is a serious complication in hemophilia A (HA) patients after FVIII replacement therapy. Inhibitors render regular prophylaxis ineffective and increase the risk of morbidity and mortality. Immune tolerance induction (ITI) regimens have become the only clinically proven therapy for eradicating these inhibitors. However, this is a lengthy and costly strategy. For HA patients with high titer inhibitors, bypassing or new hemostatic agents must be used in clinical prophylaxis due to the ineffective ITI regimens. Since multiple genetic and environmental factors are involved in the pathogenesis of inhibitor generation, understanding the mechanisms by which inhibitors develop could help identify critical targets that can be exploited to prevent or eradicate inhibitors. In this review, we provide a comprehensive overview of the recent advances related to mechanistic insights into anti-FVIII antibody development and discuss novel therapeutic approaches for HA patients with inhibitors.

## Introduction

Hemophilia A (HA) is an X-linked recessive genetic bleeding disorder caused by a deficiency of coagulation protein factor VIII (FVIII). If patients with severe HA do not undergo appropriate prophylaxis, they can suffer from spontaneous bleeding episodes and chronic damage to soft tissues, joints, and muscles. The latest global report from the World Federation of Hemophilia (WFH; compiled in 2019) indicates that the current prevalence of HA is 23.2 in 100000 live male births (1). The clinical phenotype and severity of bleeding in HA patients are generally correlated with residual FVIII levels: mild (5-40% FVIII), moderate (1-5%), or severe (< 1%) (2).

Current treatment approaches for HA involve intravenous infusion of clotting FVIII, which is the mainstay for hemophilia therapy. However, up to 30% of patients with severe HA develop inhibitors, rendering treatment with regular replacement factor concentrates ineffective and increasing morbidity and mortality rates (3). Several genetic and non-genetic factors could be involved in forming inhibitors, and there is currently no clear biomarker to predict whether a patient will produce FVIII inhibitors (4). In 1977, immune tolerance induction (ITI) regimens were first proposed for the treatment of HA patients with inhibitors. However, this regimen requires the frequent administration of relatively large doses of FVIII to downregulate the established immune response and induce immune tolerance to FVIII (5). Since then, this strategy has been modified, including variations in the FVIII dosage and product type, and combinations of additional immunosuppressive agents have been developed. The three most famous protocols are the Bonn (high-dose), Van Creveld (low-dose), and Malmo (refractory patients) (6). Of note, the ITI protocol is the only widely accepted method for establishing FVIII immunologic tolerance. Current consensus guidelines recommend that ITI should be first attempted in patients with FVIII inhibitors (7–9). However, ITI administration is generally provided *via* frequent central venous catheter infusion, which could increase the risks of thrombosis and infection (10). Moreover, approximately 30% of HA patients cannot achieve sustained immune tolerance to FVIII (11, 12). It remains unclear why some patients never trigger an anti-FVIII immune response, while others do. As such, the prevention and/or eradication of inhibitors to FVIII is important for managing HA.

The purpose of this review is to highlight the current concepts regarding the underlying pathogenesis of FVIII inhibitor development and the therapeutic advances in HA patients with FVIII immunity.

## Pathophysiology of FVIII inhibitor development

### Characteristics of FVIII inhibitors

FVIII inhibitors are polyclonal IgG antibodies with high affinity, among which IgG4 is typically a major component (13). Most inhibitors neutralize the procoagulant activity of FVIII through steric hindrance, and the most common target epitopes are the A2, A3 and C2 domains of FVIII, which prevent interaction with factor IIa, factor IXa, factor X, von Willebrand factor (VWF), and phospholipids (14). Lacroix-Desmazes et al. (15) found that an additional mechanism of anti-FVIII antibodies is hydrolysis. In 50% of inhibitor-positive patients, anti-FVIII antibodies have enzymatic activity and affect the procoagulant function of FVIII through catalytic hydrolysis of multiple FVIII cleavage sites. This type of anti-FVIII antibody can be clinically detected by the Bethesda or modified Nijmegen method. The titer is reported in the Bethesda units/ml (BU/ml). A high titer inhibitor is defined as an inhibitor level ≥ 5 BU/ml, and a low titer inhibitor is defined as a level < 5 BU/ml (3). Low-titer inhibitors comprise 25% to 50% of observed inhibitors (16), and standard alternative therapies are available for these patients (3). However, by-passing agents are required to treat bleeding episodes in patients with high-titer inhibitors (3, 16).

On the other hand, a few FVIII isoantibodies do not directly inhibit the coagulation activity of FVIII but bind to FVIII antigen epitopes to form circulating immune complexes that are cleared by the reticuloendothelial system, accelerating the metabolic processing of FVIII, shortening the half-life of the internal and external sources of FVIII, and affecting the efficacy of clinical treatment. In this regard, a competition-based enzyme-linked immunosorbent assay (ELISA) could help to differentiate neutralizing FVIII inhibitors from non-neutralizing FVIII-specific antibodies (NNAs) (17). However, as the class of NNAs has been detected in both healthy individuals and PUPs, the true clinical significance of NNAs remains unknown (18, 19). Cannavo et al. reported that the presence of NNAs substantially increased the risk of inhibitor development and that detection of NNAs could be an early predictor of inhibitor development (20). These reports provide further rationale for monitoring NNAs in HA patients. Of note, IgG4 has been identified as the most abundant IgG subclass in HA patients with FVIII inhibitors, though FVIII-specific IgG4 is absent in patients without FVIII inhibitors and in healthy subjects (13). The results from the Hemophilia Inhibitor Previously Untreated Patients (PUPs) Study (HIPS) demonstrated that the development of FVIII inhibitors within the first 50 exposure days (EDs) is associated with distinct antibody signatures (21). Patients with persistent FVIII inhibitors develop unique signatures of FVIII-binding IgG1, followed by IgG3 and IgG4. Low-affinity IgG1 maturing into high-affinity IgG1, IgG3, and IgG4 is correlated with the development of neutralizing FVIII-specific antibodies. High-affinity IgG4 can only be detected after or at the same time as the first FVIII inhibitor detection (21). The distinct antibody signatures could serve as candidates for early biomarkers of FVIII inhibitor development.

### Risk factors for inhibitor development in severe HA

Inhibitor development is a complex and multifaceted process involving interactions between genetic (unchangeable) and nongenetic (changeable) factors. The former category includes *F8* gene mutations, single nucleotide polymorphisms at the HLA locus, mutations in the immunomodulatory gene, race and family history; the latter category includes the age of first exposure, treatment intensity, type of FVIII preparation, presence of infection and history of surgery (22). Genetic factors are the basis and a prerequisite for the production of inhibitors, while nongenetic factors are typically the triggering factors. Both genetic and nongenetic factors mediate the development of inhibitors and determine their severity and duration.

Severe HA patients with large deletions, nonsense mutations, or intron 22 inversions in the *F8* gene have 7-10 times as many inhibitors as those with a small deletion/insertion, missense mutation, or splicing-induced mutation of this gene (23, 24). In a cohort of 203 children with severe HA with inhibitors, 196 cases (96.6%) were identified to have *F8* mutations. Of those patients, major mutation types include intron 22 inversions, nonsense mutations, and large deletions or insertions focusing on high- and medium-risk *F8* gene mutation types. The authors found that large deletions or insertions encompassing multiple exons and nonsense mutations residing in the light chain contributed to the progression to a high-titer inhibitor and higher peak inhibitor titer in people with severe HA (25). As such, it is highly recommended that gene mutation detection should be conducted for all newly diagnosed HA patients, particularly since it is important for guiding subsequent clinical treatments.

A recent study further demonstrated a significant association of variants in the human leukocyte antigen (HLA) region (26). In particular, low-frequency variants in *GRID2IP* are closely related to high-titer inhibitors. Another report highlighted that HLA class II molecules play an essential role in inhibitor formation. This report demonstrated that the high-*TNF-α/*high-*IL-10* genotype is associated with an increased risk of immune response to FVIII in severe HA (27). Although certain cytokine genetic polymorphisms have been implicated in inhibitor development, these results are inconsistent between populations with various genetic backgrounds. To further understand the roles of innate immune cells and mechanisms of inhibitor development versus immune tolerance, achieved with or without ITI therapy, researchers performed temporal transcriptomics profiling for HA subjects with and without a current or historic inhibitor using RNA-Seq. HA subjects with a current inhibitor showed differential expression of 56 genes and a clustering analysis identified three major temporal profiles. Interestingly, Gene Ontology (GO) enrichments featured innate immune modulators, including *NLRP3, TLR8, IL32, CLEC10A*, and *COLEC12. NLRP3* and *TLR8* are associated with enhanced secretion of the pro-inflammatory cytokines IL-1β and TNFα, while *IL32* is associated with both inflammatory and regulatory immune processes. The inflammatory status of HA patients suffering from an ongoing inhibitor includes up-regulated innate immune modulators, which could negatively affect the responses and outcomes of ITI therapy (28).

Nongenetic risk factors for the occurrence of inhibitory antibodies are largely related to FVIII treatment. Lorenzo and colleagues reported that receiving initial treatments at a young age increase the incidence of FVIII inhibitor development in patients with severe HA (29). The other report confirmed that the risk of high inhibitor formation significantly increases in HA children by early exposure to recombinant FVIII (rFVIII) concentrates (30). In this report, the immunogenicity of plasma-derived FVIII (pdFVIII) is speculated to be weaker than that of rFVIII due to VWF protection (30). Multiple studies support that VWF protects FVIII from protease degradation and attenuates FVIII memory immune responses (31, 32). Indeed, the latest evidence from a global multicenter randomized controlled clinical trial (33, 34) showed that for PUPs, the incidence of inhibitors among those treated with rFVIII preparations was markedly higher than in those treated with pdFVIII preparations. This study also stressed that rFVIII should be avoided in the early stages of treatment. To reduce the incidence of inhibitors, the differences in the immunogenicity of the two FVIII preparations require further investigation.

The retrospective CANAL study investigated 366 PUPs with severe HA from 14 European and Canadian hemophilia treatment centers (35). The results demonstrated that the risk of inhibitor development could be explained by early, intensive treatment with FVIII. Intensive FVIII treatment caused by major bleeds or surgeries could become an independent risk factor for inhibitor development. Additionally, early, regular prophylaxis could protect patients with hemophilia against the development of inhibitors (35). Moreover, a large, international cohort study (RODIN study) further demonstrated that high-dose intensive FVIII treatments increase the risk of inhibitor development in PUPs with severe HA (36). The risk of inhibitor development is highest during the first 10 to 20 EDs and decreases to less than 1% after 50 EDs. Prophylactic FVIII treatment reduces inhibitor risk, especially in patients with low-risk *F8* mutations (36). However, identifying those patients who could benefit from prophylaxis is challenging and should be addressed in additional studies.

### Risk factors associated with inhibitor development in nonsevere HA

In contrast to severe HA, patients with moderate/mild HA have a life-long risk of inhibitor development. Both genetic and environmental factors influence the risk of inhibitor development in patients with nonsevere HA (37–39). The type of *F8* mutations is a key risk factor for the occurrence of inhibitors, in patients with severe HA or nonsevere HA (40). In the INSIGHT study, the authors observed that nonsevere HA patients with splice-site mutations had a similar risk as patients with intron 22 inversions. This could be due to the effect of conserved nucleotide positions, which had a higher risk of inhibitor development than those in nonconserved nucleotide positions. The mutations associated with inhibitors in the INSIGHT cohort were all located within regions encoded for the light chain and the A2 domain of FVIII (41). This study further showed that the risk of inhibitor development was 6.7% at 50 ED and increased to 13.3% at 100 ED (41), which differs from the cases of severe HA mentioned above (36). As such, inhibitors should be routinely screened during the time of greatest risk for inhibitor development in patients after FVIII replacement therapy. In pediatric patients, inhibitor screening should be performed every 5 EDs for the first 20 EDs after the first treatment with clotting factor products (36, 40, 42, 43).

Inhibitors usually occur at an earlier age in nonsevere HA. Recently, a total of 6624 persons with nonsevere HA were investigated for an average of 8.5 years in the United States (38). The results demonstrated that the prevalence of inhibitors was 2.6% (n = 171), occurring at a median age of 13 years. However, the occurrence of inhibitors at an early age was not associated with increased mortality. Previous studies revealed that older age is another risk factor for inhibitor development (40, 44). Age-related immune dysregulation and late loss of tolerance could be particularly relevant for the nonsevere hemophilia cohort, as exposure to therapeutic FVIII concentrates is distributed throughout a lifetime and often skewed toward later decades for elective operative interventions. Moreover, both high-dose FVIII treatment and surgical interventions could increase the risk of inhibitor development in nonsevere HA patients (39, 45). In the INSIGHT study, the all-cause mortality rate in nonsevere HA patients with inhibitors was more than five times higher compared to those without inhibitors (46). Inhibitor-related mortality primarily increased in older patients with high inhibitor titers. These findings indicate that nonsevere hemophilia is not mild and highlight the importance of closely following-up with these patients.

Inhibitor occurrence connected with the surrounding microenvironment has recently raised concern. Researchers have found that inhibitors are often produced along with the surrounding danger signals. The “danger theory”, proposed by Polly Matzinger in 1994 (47–49), postulated that the human immune response is triggered by “danger signals” or “alarm signals”. Patients are often exposed to various endogenous or exogenous danger signals, such as those resulting from surgery, trauma, and infection, during treatment with FVIII. These danger signals, termed danger-associated molecular patterns (DAMPs), could become the major drivers to elicit an anti-FVIII immune response (48, 50–54). A clinical trial reported by Kurnik et al. (55) demonstrated that the production of FVIII inhibitors decreases during the administration of the first dose of FVIII by avoiding proinflammatory stimuli (bleeding, infection, surgery, vaccination, *etc.*). However, it remains difficult to predict the potential risks associated with individual patients’ inhibitor development. The available evidence is scant and inconclusive, and distinguishing the contribution of peak treatment from danger signals remains challenging. However, by clearly identifying danger signals, we could further explore the precise mechanisms underlying the risk effect, identify different biomarkers, and provide new targets to effectively prevent inhibitor development. As such, in the future clinical management of HA patients, the impacts of danger signals should be monitored in various risk scenarios, and therapeutic strategies can be improved by avoiding unnecessary risks.

### Immunological pathophysiology of inhibitor development

Previous studies confirmed that FVIII inhibitor development is CD4^+^ T cell-dependent in both murine models and patients (56–60). T follicular helper cells (TFHs), a novel subset of CD4^+^ T cells, work with cognate follicular B cells to trigger a germinal center (GC) reaction that is ultimately responsible for the production of anti-FVIII antibodies in HA mice (58). In a multicenter study, significant reductions in levels or even complete elimination of FVIII inhibitors were observed in HA patients with high titers of inhibitors and concomitant HIV infection (61). Mechanistically, it is the antigen-presenting cells (APCs) that present FVIII peptides on major histocompatibility complex class II (MHC-II) to T cells, triggering FVIII immune responses. Indeed, several cell types, such as dendritic cells (DCs), macrophages, B lymphocytes and endothelial cells, could act as APCs that mediate FVIII-specific immune response (59, 62). Low-density lipoprotein receptor-related protein (LRP, CD91), the mannose receptor (MR), and membrane-bound heparan sulfate proteoglycans (HSPGs) play important roles in FVIII endocytosis by binding to the A2, A3 and C1 domains of FVIII (63–65), anti-FVIII C1 domain antibodies can prevent the endocytosis of FVIII by APCs (66). These *in vitro* and *in vivo* mouse model studies facilitate the discovery of the key factors involved in inhibitor development.

An *in vitro* study demonstrated that VWF reduced the endocytosis of FVIII by human DCs through steric hindrance effects (67). In murine HA models, researchers found that phosphatidylserine (PS) induced immune tolerance and reduced the production of inhibitors by affecting the maturation of DCs, inducing the generation of Tregs, and inhibiting the generation of memory B cells (68–70). More recently, studies have demonstrated that the binding of FVIII to lysophosphatidylserine (Lyso-PS) to form the Lyso-PS-rFVIII Fc complex significantly reduced inhibitor development in HA mice after either intravenous or oral administration of Lyso-PS (71, 72). In addition, researchers found that site N2118, containing high-mannose glycans, have a significant impact on FVIII immunogenicity in the murine HA model (73). Results from these studies can help identify valuable targets to reduce FVIII immunogenicity.

The spleen is the predominant organ involved in initiating the immune response. In a murine model study, researchers confirmed that APCs in the marginal zone (MZ) of the spleen play critical roles in the immune response to FVIII (74). Zerra and coworkers found that transfused FVIII was located in the marginal sinus of the spleen and colocalized with the B cells in the MZ of the HA mouse spleen, and the development of FVIII inhibitors could be completely blocked by specific removal of the MZ B cells (75). Moreover, the activation of APC costimulatory signals (CD40, CD40L, CD80/CD86, and CD28) is necessary for the complete activation of T cells. Indeed, blocking the CD40/CD40L interaction has been found to induce long-term immune tolerance in HA mice. Unfortunately, treatment with anti-CD40 can also activate platelets and increase the risk of thrombosis (76).

Activated T cells can differentiate into effector T cells (Teffs) and regulatory T cells (Tregs). Teffs are responsible for initiating and maintaining an effective anti-FVIII immune response by activating B cells to differentiate into mature plasma cells through a GC process. In contrast, Tregs could act as potent suppressors of the Teffs to inhibit antibody development. There is growing evidence that the generation of Tregs *via* either human samples or animal models could lead to FVIII tolerance, suggesting that the balance between Teffs and Tregs is another key determinant involved in the pathophysiology of FVIII inhibitor development (57, 77–80). A recent study showed that immune tolerance against FVIII under non-hemophilic conditions was maintained by programmed death (PD) ligand 1 (PD-L1)-expressing Treg (81). In addition, B cell-activating factor (BAFF) cytokine family is a key regulator of B cell differentiation in normal homeostasis and immune disorders. A recent study demonstrated that BAFF levels are elevated in pediatric HA inhibitor patients and in those who failed to achieve immune tolerance with anti-CD20-mediated B cell depletion. Using a mouse model, the authors found that BAFF modulated tolerance induction and inhibitor eradication by downregulating plasma cells, demonstrating the important role of BAFF in the modulation of anti-FVIII response (82).

Moreover, memory B and/or long-lived plasma cells (LLPCs) play a key role in maintaining established anti-FVIII immune responses. Importantly, FVIII-specific memory B cells are present in HA patients with inhibitors, while these cells are absent in healthy controls or patients without inhibitors (83). Following re-exposure to FVIII, the unique memory B cells can rapidly differentiate into plasma cells that produce high levels of anti-FVIII antibodies (84). Additionally, there is evidence that memory B cells contribute to replenishing the pool of LLPCs in the bone marrow (BM) (85). GC reaction memory B cells can develop into plasma blasts that migrate to the BM, and mature into LLPCs that maintain serum levels of antibodies for prolonged periods. LLPCs persistently producing anti-FVIII antibodies in BM are unlikely to be affected by conventional high dosages of FVIII infusions (84, 86). Therefore, for long-term induction FVIII tolerance, it is highly necessary to develop novel strategies targeting FVIII-specific memory B cells and LLPCs.

## Novel strategies for treating HA with inhibitors

### Conventional hemostatic bypassing agents

The development of FVIII inhibitors in HA patients remains challenging in clinical management. Currently, the mainstay of treating HA inhibitor patients requires controlling bleeding symptoms and eliminating alloantibodies against FVIII. Unfortunately, ITI protocols are not always effective in patients with high levels of inhibitors (87–89), making alternative strategies clinically necessary. In recent years, promising therapeutic strategies have been identified, including treatment with immunomodulatory drugs or molecules, oral or transplacental delivery of FVIII, and cell or gene therapy (90–92).

For patients who appear to be ineffective for standard replacement therapies and have confirmed inhibitors, a “bypass approach” should be adopted immediately to stop bleeding regardless if the patients undergo ITI or not (9); this approach includes treatment with recombinant activated factor VII (rFVIIa) and activated prothrombin complex concentrate (aPCC). Either rFVIIa or aPCC could be used to treat bleeding episodes (BEs) in both HA and hemophilia B (HB) patients with inhibitors (9). However, a major concern remains about the risks of thrombotic microangiopathy in aPCC-treated patients receiving non-factor prophylactic agents such as emicizumab (93). In addition, bypassing agents are expensive and provide incomplete hemostatic correction in some patients (94). As such, new strategies for the control and prevention of BEs are highly anticipated. Various new approaches based on non-factor replacement therapies have been developed and entered clinical trials, which include facilitating the coagulation pathway (*e.g.* emicizumab) and blocking the anticoagulant pathway (*e.g.* concizumab, fitusiran).

### Emicizumab: Bispecific monoclonal antibody

The new hemostatic agent emicizumab is a humanized bispecific monoclonal antibody that binds to and bridges activated factor IX (FIXa) and factor X to simulate the physiological function of FVIII (95). It has been licensed for bleeding prophylaxis in HA patients with or without inhibitors, and is the first non-factor replacement therapy for HA (93). Subjects enrolled in early clinical trials tolerated once-weekly subcutaneous injections of emicizumab at various doses (0.3, 1.0, and 3.0 mg/kg); no bleeding occurred in 73% of subjects, and no neutralizing antibodies specific for emicizumab were observed during 12 weeks of treatment in the early phase of clinical trial (96, 97).

Data from the HAVEN 1 phase 3 trial showed that among the 109 HA patients aged 12 years and older with high titers of inhibitors, the bleeding rate in the trial group treated with emicizumab was 87% lower than in the untreated group (95, 98). Emicizumab significantly improved the quality of life of patients and had an obvious effect in preventing bleeding. The HAVEN 2 study enrolled 88 children with inhibitors with a median age of 7 years (range, 1-15 years) (99). In this trial, participants who received an emicizumab injection with 4 once-weekly loading doses of 3 mg/kg followed by a maintenance regimen of once weekly (1.5 mg/kg, QW) showed a low annualized rate of treated bleeding event (ABR), with 77% of participants experiencing no treated bleeding events. Notably, efficacy was maintained among those who received emicizumab every 2 weeks (3 mg/kg, Q2W) or every 4 weeks (6 mg/kg, Q4W). This large prospective study highlighted the special role of emicizumab as a highly effective novel medication for pediatric HA patients with anti-FVIII inhibitors.

The efficacy, safety, and pharmaceutics of these treatments have been further studied in the phase 3 HAVEN 3-5 studies, which included adults/adolescents ≥ 12 years of age with and without inhibitors (100–102). These trials demonstrated that emicizumab prophylaxis achieved remarkable efficacy for bleeding control and was well tolerated in HA patients, regardless of FVIII inhibitor status. Of note, the long-term efficacy and safety of emicizumab for up to 5.8 years were reported in patients with severe HA (103). Nevertheless, emicizumab provides more convenient and feasible administration way (maintenance dose of once every 1, 2 or 4 weeks) than conventional FVIII replacement therapy. No anti-FVIII antibodies developed among those participants during administered emicizumab, and neither serious nor thrombotic adverse events were reported in either of these clinical trials (104). However, as thrombosis and thrombotic microangiopathy occurred in some participants who had received aPCC in HAVEN 1 (95), WFH recommends the use of rFVIIa instead of aPCC for treating breakthrough bleeding during emicizumab prophylaxis (9). Moreover, 0.33% (25/7500) of subjects produced an immune response to emicizumab (93). In the HAVEN trials, 2 out of 88 pediatric participants in HAVEN 2 (99, 105) and 1 out of 64 evaluable participants in HAVEN 5 (102) developed anti-drug antibodies (ADAs) with neutralizing potential. Although this occurs infrequently, it is necessary to monitor the neutralizing ADAs to implement immunological surveillance in HA patients with emicizumab regimens.

The final analysis of the STASEY phase III clinical trial enrolled a total of 193 HA patients age ≥ 12 years with inhibitors who received emicizumab once weekly for up two years (106). The results revealed that emicizumab was well-tolerated in the cohort of participants. The most common AEs were arthralgria (17.1%), naspharyngitis (15.5%), and headache (15%). Emicizumab-related AEs were observed in 18.1% of participants, with injection site reactions being the most frequent (9.8%). No emicizumab-related thrombotic microangiopathies (TMAs) or thromboembolic events (TEs) were reported in five participants who also received aPCC. ADAs were found in 5.2% of participants, 2.6% of whom were classified as having ADAs that were neutralizing *in vitro*. Most importantly, emicizumab continued to present effective bleeding control, with 82.6% of participants achieving no bleeding episodes that required treatment. The final analysis of the phase IIIb STASEY study further confirmed the favorable safety profile of emicizumab, which is consistent with the phase III HAVEN clinical program.

The first case of an emicizumab ADA was reported in clinical trials (107). In this report, a 6-year-old boy with severe HA with inhibitors received standard loading doses of emicizumab and was then administered a 1.5 mg/kg/week regimen. However, 3 months into treatment the patient presented with a traumatic bleed due to the loss of emicizumab effect with a proven ADA. Emicizumab was discontinued and the patient resumed daily prophylaxis with rFVIIa. Therefore, it is important for clinicians to be aware that meaningful ADAs can occur, and to refer the patient to a special coagulation laboratory for ADAs testing if clinical suspicion arises. Moreover, real-world data from 52 pediatric patients with severe HA revealed that emicizumab was safe and well tolerated and that minor AEs, including headaches, abdominal pain and nausea, and injection site reactions, occurred in about 7.7% of patients. Four patients experienced major AEs, including severe headaches, major bleeding events, development of ADAs, and recurrence of inhibitors. Emicizumab prophylaxis was discontinued in three patients (5.7% of the cohort) due to AEs. No AEs were reported in four PUPs of the cohort (108).

Although emicizumab demonstrated significant reductions in ABR compared with standard prophylaxis, however, bypassing agents, such as aPCC, should be used with caution. As mentioned above, 3 patients developed TMA in HAVEN 1 and 2 additional TEs were reported among 401 participants in HAVEN 1-4 (109). For the 3 patients who developed TMA, one proposed explanation is that these patients concomitantly received aPCC, which contains the targets of emicizumab, FIX/IXa and FX/Xa. The excess substrate availability could have resulted in uncontrolled thrombin generation and subsequent thrombotic complications.

Currently, there are other FVIII mimetics under development. Mim8 is a next-generation bispecific to FIXa and FX, which shows enhanced thrombin generation *in vivo* compared with a sequence-identical analog of emicizumab and an extended half-life of 14 days in cynomolgus monkeys (110). Subcutaneous administration of Mim8 up to 3 mg/kg/week for 26 weeks resulted in relevant pharmacodynamic effects, with no signs of thrombi or excessive coagulation activation (111). The FRONTIER 1 study is an ongoing phase 1/2 study of Mim8 in the HA participants with or without inhibitors ≥12 years of age. Another FVIII mimetic bispecific antibody, BS-027125, is currently in preclinical evaluation with no human data yet available.

### Targeting natural anticoagulants

In recent years, novel non-factor replacement therapies based on targeting of natural anticoagulants have been acknowledged; and the efficacy and safety in HA patients with inhibitors are currently ongoing in clinical trials. These new strategies consist of targeting negative clotting regulators such as tissue factor pathway inhibitor (TFPI), antithrombin (AT), or activated protein C (aPC).

TFPI presents two splicing forms TFPIα and TFPIβ. TFPIα is a soluble form of TFPI that is responsible for inhibiting prothrombin activity in the extrinsic coagulation system. It inhibits FVIIa *via* its K1 domain, FXa *via* its K2 domain, and protein S *via* its K3 domain (112). Current research focuses on monoclonal antibodies (mAbs) specific to the K2 domain. Studies on the evaluation of these novel molecules, including befovacimab, concizumab, and marstacimab, have been ongoing in participants with HA or HB, regardless of the presence of inhibitors. Of these, befovacimab also targets the K1 domain of the TFPI (113). While favorable results were obtained from the preclinical *in vivo* studies and phase 1 clinical trials, the phase 2 study was terminated early due to three befovacimab-related thrombotic serious adverse events (SAEs) (114). Therefore, the therapeutic window of anti-TFPI treatment must be further investigated.

Concizumab is an IgG4 humanized mAb selectively targeting the K2 domain of the TFPI. Results from phase 1-3 clinical trials in healthy volunteers and HA patients showed a dose-dependent increase in D-dimer and thrombin fragments after concizumab treatment, accompanied by significant hemostatic efficacy and tolerability. These results indicate the adequate safety and half-life characteristics of concizumab, whether administered subcutaneously or intravenously (115–117). However, in phase 3 of the clinical trial, three patients experienced five nonfatal thrombotic SAEs (118, 119). These AEs were acute myocardial infarction in one patient with HA, a renal infarction in one HB patient with inhibitors, and three TEs (deep venous thrombosis, pulmonary embolism, and superficial thrombosis of the vein) in one patient with HA. This trial had to be temporarily suspended and resumed after modifying the protocols for treating breakthrough bleeding. Shapiro and colleagues presented the results of concizumab prophylaxis from the extension parts of phase 2 explorer 4 (HA or HB with inhibitors) and explorer 5 (severe HA without inhibitors) trials (119). The long-term efficacy of concizumab was maintained during the trial, and estimated ABRs at the last concizumab dose after more than 76 weeks of treatment were comparable to those observed in the main parts (≥ 24 weeks). Although the authors found that around 25% of patients developed ADAs, the ADAs levels were low and transient, with no observed SAEs in most cases. Phase 3 trials are currently ongoing, and will provide further insight into the efficacy and safety of concizumab to treat hemophilia in the future. Moreover, other humanized mAbs, such as marstacimab, which specifically binds to the K1 domain of the TFPI, have shown the feasibility and efficacy of targeting TFPI therapies in hemophilia. A phase 1b/2 clinical study demonstrated clinically meaningful reductions in ABRs and treatment-related changes for all pharmacodynamics biomarkers across all marstacimab dose levels in participants with hemophilia (120). Together, therapeutic advances in hemophilia with targeting TFPI treatments are promising. Currently, two of the three novel anti-TFPI mAbs have progressed to phase 3 clinical trials. However, the potential thrombotic issue remains a key consideration and should be thoroughly evaluated in ongoing clinical trials.

AT is a member of a small protein family produced in liver cells and is an effective thrombin inhibitor. Fitusiran is an antithrombin RNA interference molecule that can silence AT mRNA expression in liver cells, increase the production of thrombin, and reduce bleeding tendency (121, 122). Fitusiran-induced reduction of AT levels can rebalance hemostasis in patients with HA or HB, with or without inhibitors. The phase 1 dose-escalation study enrolled 25 patients with moderate to severe HA or HB (122). The results demonstrated a fitusiran dose‐dependent mean maximum AT reduction of 70%–89% from baseline, with a reduction of > 75% enabling a similar degree of thrombin generation compared with healthy volunteers. One participant reported severe chest pain, and although thrombosis was ruled out, this event led to treatment discontinuation. No ADAs developed in the enrolled four healthy volunteers or the participants with hemophilia during the study course (122, 123). The results from the phase 2 and phase 3 clinical trials showed that the thrombin peak height in the enrolled participants was related to the degree of AT reduction when fistusiran was subcutaneously administered each month. AT reduction by ≥ 75% from baseline led to a significant improvement in thrombin generation and decreased bleeding frequencies (124, 125). The SAE was a fatal cerebral sinus thrombosis in one patient during the phase 2 trial, which occurred after repeated infusions of high FVIII concentrate doses over more than 24 hours. Three additional nonfatal vascular thrombotic events occurred in late phase 3 trials despite adherence to breakthrough bleed management guidelines. These were attributed to AT levels decreasing below 10% (126, 127). As a result, studies targeting higher AT levels have been resumed, seeking to maintain a favorable benefit-risk balance for patients. Overall, fitusiran could offer either HA or HB patients with inhibitor effective prophylaxis delivered through monthly, low volume, fixed-dose and subcutaneous administration.

In general, the aim of all therapeutic strategies in hemophilia is to increase the amount of thrombin generation at sites of vascular damage, which can be generally achieved by replacing missed clotting factors or by increasing the concentration of either the enzymes or substrates that result in the production of thrombin. Aymonnier and colleagues reported a novel approach consisting of targeting a natural and potent thrombin inhibitor, named protease nexin-1 (PN-1) (128). The authors showed that a PN-1-neutralizing antibody could significantly shorten the thrombin burst in response to tissue factor in platelet-rich plasma (PRP) from mild or moderate hemophilia patients. In contrast, neutralized PN-1 did not improve thrombin generation in PRP from severe hemophilia patients. However, with collagen-induced platelet activation, PN-1 deficiency in HA mice or PN-1 neutralization in patients with severe HA resulted in significantly improved thrombin generation in PRP. Recent experimental and clinical studies revealed that serine protease inhibitors (SERPINs), *e.g.* α1-antitrypsin and SerpinPC, could decrease bleeding in hemophilia *via* selective inhibition of aPC (129–132). Nevertheless, specific inhibition of the aPC anticoagulant function by the novel inhibitory mAb has been designed and developed in recent years (133–135). Indeed, the administration of mAb selectively inhibits aPC’s anticoagulant activity, but does not compromise its cytoprotective function, and offers a better therapeutic potential alternative for HA.

## Novel strategies for eradicating inhibitors or immune tolerance induction

### AAV-liver gene therapy

In recent years, hepatic *in vivo* gene therapy *via* adeno-associated virus (AAV) vectors has demonstrated promising results in clinical trials for hemophilia patients in the first year after treatment, but FVIII levels steadily decline afterward. Compared to those in HB, clinical trials in HA have been slower due to the large size of *F8* cDNA (7 kb). Encouragingly, after removing the sequence encoding the nonfunctional domain (B-domain deletion, BDD), the truncation of *F8* can be incorporated into AAV vectors (136). The first successful application of this approach was found in 6 of 7 severe HA participants who received a single high-dose cohort of an AAV5 vector encoding a BDD *F8* (AAV5-hFVIII-SQ) (137). The FVIII activity (FVIII:C) level of these participants remained stable at or above the physiologic range 1 year after receiving AAV5-hFVIII-SQ liver-directed gene therapy. The annualized rates of FVIII concentrate use and treated bleeding significantly decreased in the participants (138). However, multiyear follow-up data from the participants receiving AAV5-hF8-SQ gene therapy showed that FVIII expression steadily declined (139). This raises concerns about the durability and safety of AAV-based liver-specific gene therapy. Currently, several AAV-based gene transfer strategies have been modified and clinical trials have been initiated, which could offer hope to improve the durability and safety of this therapy for patients with hemophilia (140–143).

To date, only adult men with endogenous factor levels ≤ 2% and without the advanced liver disease have received gene therapy. It remains unknown whether HA patients with inhibitors respond to AAV liver-directed gene therapy approach. Evidence from murine and canine models has demonstrated that liver-directed *F8* gene therapy could induce tolerance towards a primed immune system with pre-existing inhibitors (144–147). In HA dogs with pre-existing inhibitors after canine(c) FVIII AAV-based liver-directed gene therapy, there is an early increase in CD4^+^ CD25^+^ FOXP3^+^ Tregs within the first few days (145, 146). This is associated with a decline in anti-cFVIII antibodies but was not observed in HA dogs without inhibitors receiving similar therapy. Furthermore, immune tolerance was established in treated dogs even after repeated challenges with cFVIII proteins. In mouse model studies, AAV-F8 gene therapy can not only correct FVIII levels, but also results in low- to undetectable inhibitor titers following subsequent challenges with rFVIII in HA mice on a BALB/c background (147, 148). The ability to induce FVIII tolerance in these mice occurred in both the presence and absence of transient immunosuppression mediated by B cell depletion with anti-CD20. In contrast, HA mice on a mixed BL/6-129/sv background following AAV-F8 gene transfer only resulted in modest correction of the clotting time below that of untreated mice regardless of anti-CD20 treatment. These mice lost bleeding phenotype correction along with the development of inhibitors when challenged with supplementary rFVIII. However, transient B cell depletion with anti-CD20 at the time of AAV-F8 gene transfer resulted in subsequent hyporesponsiveness to FVIII with significantly lower inhibitors (147). Thus, these results highlight the potential role of genetics in determining whether a patient will respond to AAV-F8 gene therapy. Importantly, targeting B cell method could further improve tolerance induction using this novel approach.

As mentioned above, a combination regimen targeting FVIII-specific memory B cells and LLPCs facilitate ITI to FVIII has recently attracted research interest. A study by Liu et al. found that anti-CD20 combined with IL-2/IL-2 mAb complexes plus rapamycin significantly reduced both FVIII-specific antibody-secreting cells and memory B-cells in HA inhibitor mice (148). The combination had a synergistic effect on the inhibition and neutralization of antibody production. Moreover, as LLPCs play a key role in maintaining established antibodies, the same group developed a novel strategy that could target LLPCs (149). In the regimen, AMD3100, an antagonist of CXCR4, was used to block the CXCL12/CXCR4 interaction and inhibit the homing and retention of LLPCs (150). The results demonstrated that the combined treatment using AMD3100, G-CSF, anti-CD20 and IL-2/IL-2 mAb complexes effectively eradicated LLPCs and inhibitor titers in either FVIII protein- or FVIII plasmid-primed HA inhibitor mice. Encouragingly, a recent study found that B cell depletion with anti-CD20 eliminates memory B cells and enhances FVIII tolerance when combined with rapamycin, preventing neutralization of the newly expressed clotting FVIII in HA mice following AAV8-*coF8* gene therapy. In addition, it has been reported that combining rapamycin and anti-CD20 therapy with conventional FVIII ITI treatment could induce FVIII tolerance in patients with previously refractory FVIII inhibitors (151). Herein, a combination of anti-CD20/mTOR inhibition strategies could increase the success of ITI and allow for the inclusion of HA inhibitor patients for AAV-mediated *F8* gene therapy or FVIII ITI in clinical trials.

### LV-platelet gene therapy

Lentiviral vector (LV)-mediated gene therapy has demonstrated significant potential for treating FVIII deficiency. In particular, LV-mediated platelet-targeted gene transfer into hematopoietic stem cells (HSCs) results in stable integration of the *F8* gene into the host genome, leading to persistent therapeutic effects in hemophilia models with and without inhibitors (152–157). The ectopic expression of FVIII under the platelet-specific promoter control enables FVIII storage together with VWF in α-granules in platelets. This could shield FVIII from the circulating neutralizing anti-FVIII antibodies until it is delivered to the site of injury upon platelet activation. Even relatively small numbers of activated platelets that locally excrete FVIII could be sufficient to promote efficient clot formation in the recipient mice. Notably, none of the transduced mice developed an anti-FVIII immune response even when challenged with rFVIII. The inhibitor titers decreased with time in the pre-existing anti-FVIII immunity models after LV-F8 gene therapy (57, 152, 155, 157, 158). Furthermore, infusion of platelets containing FVIII triggers neither primary nor memory anti-FVIII immune responses in HA mice (92), indicating that it could become a useful alternative treatment approach for HA inhibitor patients if the availability of AAV-based gene therapy is limited by certain circumstances. Importantly, novel gene therapy strategies could also induce Tregs expansion and CD4^+^ T cell-mediated immune tolerance (155).

Since the LV-F8 gene therapy approach targets HSCs, this strategy could result in lifelong FVIII production. Encouragingly, the LV-mediated therapeutic strategy could also provide an alternative gene therapy for patients with pre-existing immunity to the AAV. Currently, a phase 1 clinical trial using an LV-mediated targeting platelet *F8* gene delivery system has been initiated at the Medical College of Wisconsin (ClinicalTrials.gov: NCT03818763) (141). However, either total body irradiation or chemotherapy plus immune suppression is required to facilitate engraftment and efficacy, which could limit widespread applications of this strategy in clinical settings.

### Nonviral delivery system

Oral tolerance induction *via* transplastomic lettuce expressing FVIII or FIX fused to a transmucosal carrier is at the forefront of hemophilia treatment research. Such bioencapsulated factors can be ingested, cross the intestinal epithelium, and induce Treg cells within the gut-associated lymphoid tissue (141). Sherman et al. demonstrated that the FVIII heavy chain and C2 domain can be expressed as cholera toxin B subunit fusion proteins in tobacco chloroplasts (159). Oral delivery of a mixture of these bioencapsulated antigens could suppress and reverse inhibitor formation in HA mice. Intestinal microbiota plays a vital role in maintaining immune homeostasis through the interaction between the microbiome and the intestinal immune system (160). A recent study revealed that tolerance induction by oral delivery of antigens bioencapsulated in plant cells occurs *via* the unique immune system of the small intestine, and the inhibition of antibody formation primarily performed by CD4^+^ CD25^−^ FoxP3^−^ LAP^+^ Tregs (but not of CD4^+^ CD25^+^ FoxP3^+^ Tregs) (161). Antigen release from the plant cells occurs *via* bacteria with required enzymatic degradation activities in the small intestine and delivery to the associated immune system. The composition of this microbiome is distinct from that of the large intestine, and their augmentation could further promote plant cell-based oral tolerance induction in the treatment of hemophilia. The ongoing advances of the novel nonviral delivery system in animal HA models make it a likely candidate for clinical trials.

Since the goal of the development of tolerogenic therapies is to suppress antibody formation, Tregs have been proposed as a potential clinical therapy for various adverse immune disorders. Adoptive transfer of *ex vivo*-expanded Tregs improved immune cell engraftment and graft-versus-host disease after HSC transplantation in the clinical trial (162). Nevertheless, chimeric antigen receptor (CAR) Tregs expansion has been identified as a novel biomarker of response and toxicity after CAR T cell therapy, which raises the prospect that this subset could regulate CAR T cell response in humans (163). Interestingly, Yoon et al. demonstrated that FVIII-specific CAR (ANS8 CAR) Tregs can be engineered, and demonstrates their ability to inhibit T- and B-cell effector responses to FVIII (164). Importantly, ANS8 CAR-transduced Tregs can suppress the recall antibody response of murine splenocytes from FVIII knockout mice to FVIII *in vitro* and *in vivo*. Notably, CAR-modified Tregs engineered in a non-MHC restricted manner have widespread applications and have gained increasing attention in recent years (165, 166). This novel cellular therapy remains a promising approach for the future tolerogenic treatment of HA patients with existing inhibitors.

## Closing remarks

Anti-FVIII immunity represents a major limitation of HA therapy. Multiple factors impact inhibitor development in patients with HA, although novel alternative therapeutic options can be used to prevent bleeding disorders in HA patients with and without harboring inhibitors. However, the benefits are only available for a small number of HA patients, and the lack of infrastructure facilitating the worldwide availability of novel strategies remains a barrier. There is also an unmet need to provide further insight into the longer-term efficacy and safety of these novel approaches. As such, mechanistic insights into humoral immunity to FVIII and identifying new biomarkers connecting inhibitor generation remain top priorities for clinical or preclinical investigations.

## Author contributions

YC wrote, revised, edited, critically reviewed and submitted the manuscript. LL drafted part of the manuscript. QZ, ZC and MH provided comments and discussions for the manuscript. LF and JH critically reviewed the manuscript. QS critically reviewed, edited, and revised the manuscript. All authors contributed to the article and approved the submitted version.

## Glossary

**Table:**

HA	Hemophilia A
FVIII	Factor VIII
WFH	World Federation of Hemophilia
ITI	Immune tolerance induction
Teffs	Effector T cells
Tregs	Regulatory T cells
VWF	von Willebrand factor
NNAs	Nonneutralizing antibodies
ELISA	Enzyme-linked immunosorbent assays
HLA	Human leukocyte antigen
GO	Gene Ontology
rFVIII	Recombinant FVIII
pdFVIII	Plasma-derived FVIII
PUPs	Previously untreated patients
HIPS	Hemophilia Inhibitor Previously Untreated Patients Study
EDs	Exposure days
BU	Bethesda units
ISTH	International Society of Thrombosis and Hemostasis
DAMPs	Danger-associated molecular patterns
APCs	Antigen presenting cells
DCs	Dendritic cells
rFVIIa	Recombinant activated factor VII
aPCC	Activated prothrombin complex concentrate
BEs	Bleeding episodes
HB	Hemophilia B
AEs	Adverse events
GC	Germinal center
BAFF	B cell-activating factor
LLPCs	Long-lived plasma cells
BM	Bone marrow
MHC-II	Major histocompatibility complex class II
LRP	Lipoprotein receptor-related protein
HSPGs	Heparan sulfate proteoglycans
PS	Phosphatidylserine
Lyso-PS	Lysophosphatidylserine
MZ	Marginal zone
ADAs	Anti-drug antibodies
TMAs	Thrombotic Microangiopathies
TEs	Thromboembolic events
QW	Once weekly
TFPI	Tissue factor pathway inhibitor
AT	Antithrombin
aPC	Activated protein C
mAb	Monoclonal antibody
SAE	Serious adverse event
AAV	Adeno-associated virus
LV	Lentiviral vector
BDD	B-domain deletion
ABR	Aannualized rate of bleeding events
PN-1	Protease nexin-1
PRP	Platelet-rich plasma
SERPINs	Serine protease inhibitors
FVIII:C	FVIII activity
HSC	Hematopoietic stem cell
CAR	Chimeric antigen receptor
